# Differential spatial responses of rodents to masting on forest sites with differing disturbance history

**DOI:** 10.1002/ece3.7955

**Published:** 2021-08-13

**Authors:** Frederik Sachser, Mario Pesendorfer, Georg Gratzer, Ursula Nopp‐Mayr

**Affiliations:** ^1^ Department of Forest‐ and Soil Sciences Institute of Forest Ecology University of Natural Resources and Life Sciences Vienna Austria; ^2^ Department of Integrative Biology and Biodiversity Research Institute of Wildlife Biology and Game Management University of Natural Resources and Life Sciences Vienna Austria

**Keywords:** *Apodemus* spp., habitat selection, masting, *Myodes glareolus*, natural disturbances, primeval forest

## Abstract

Mast seeding, the synchronized interannual variation in seed production of trees, is a well‐known bottom‐up driver for population densities of granivorous forest rodents. Such demographic effects also affect habitat preferences of the animals: After large seed production events, reduced habitat selectivity can lead to spillover from forest patches into adjacent alpine meadows or clear‐cuts, as has been reported for human‐impacted forests. In unmanaged, primeval forests, however, gaps created by natural disturbances are typical elements, yet it is unclear whether the same spillover dynamics occur under natural conditions. To determine whether annual variation in seed production drives spillover effects in naturally formed gaps, we used 14 years of small mammal trapping data combined with seed trap data to estimate population densities of *Apodemus* spp. mice and bank voles (*Myodes glareolus*) on 5 forest sites with differing disturbance history. The study sites, located in a forest dominated by European beech (*Fagus sylvatica*), Norway spruce (*Picea abies*), and silver fir (*Abies alba*), consisted of two primeval forest sites with small canopy gaps, two sites with larger gaps (after an avalanche event and a windthrow event), and a managed forest stand with closed canopy as a control. Hierarchical Bayesian N‐mixture models revealed a strong influence of seed rain on small rodent abundance, which were site‐specific for *M. glareolus* but not for *Apodemus* spp. Following years of moderate or low seed crop, *M. glareolus* avoided open habitat patches but colonized those habitats in large numbers after full mast events, suggesting that spillover events also occur in unmanaged forests, but not in all small rodents. The species‐ and site‐specific characteristics of local density responding to food availability have potentially long‐lasting effects on forest gap regeneration dynamics and should be addressed in future studies.

## Graphical abstract

We present novel hierarchical Bayesian N‐mixture models of unique count data from 14 years of small mammal live trapping investigating functional responses of dominant rodent taxa to different habitat types and mast seeding, including sites of natural disturbance events and sites in the largest remaining primeval forest of the Alps.

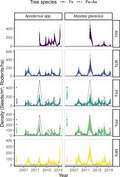

## 1 INTRODUCTION

Rodents are important seed predators and seed dispersers in temperate European forests (Kempter et al., 2018; Nopp‐Mayr et al., 2012; Ouden et al., 2005), and their population dynamics play a pivotal role for both spatial and temporal aspects of seed fate and plant recruitment in and around newly formed gaps (Hulme & Kollmann, 2005). Superabundance of food, as observed after large seed production events, positively influences overwinter survival and reproduction in small mammals (Flowerdew et al., 2017; Jensen, 1985; Johnsen et al., 2017), leading to high population numbers during subsequent time periods. As rodents´ population densities increase, reduced habitat selectivity and population spillover into forest gaps might be observed (Ecke et al., 2002; Horne, 1983; Zwolak et al., 2016) inducing changes in rodent community composition and related effects on plant recruitment. Here, we investigate how varying seed rain alters site selection of granivorous animal populations.

While bottom‐up drivers affect overall abundance in many small mammal species, population density in turn might affect habitat selection of individual species due to inter‐ and intraspecific competition (Horne, 1983; Sundell et al., 2012; Zwolak et al., 2018). Therefore, local density during periods of high overall density might not reflect average habitat preferences of a species (Horne, 1983). For example, bank voles *Myodes glareolus* typically avoid open habitats such as clear‐cuts (Bogdziewicz & Zwolak, 2014; Hansson, 1996) and alpine meadows (Zwolak et al., 2018), preferring forests with a closed‐canopy cover unless overall population density is high (Sundell et al., 2012). These temporal patterns of abundance suggest that open habitats provide suboptimal conditions for *M. glareolus* compared with adjacent forest sites (Fretwell & Lucas, 1969).

Rodent population dynamics in forest patches can be expected to differ fundamentally with the scale and frequency of disturbance events (e.g., frequent small‐scale events versus. rare larger‐scale events) and between primeval and managed forest stands. For example, Carey and Johnson (1995) found that species composition of small mammal communities in old‐growth forests was similar to managed younger forest stands (35–79 years old), while abundance was higher. An apparent difference between anthropogenic disturbances (e.g., logging activities) and natural disturbance events (e.g., uncleared windthrows) arises from the supply of remaining coarse woody debris that provides shelter for small mammals (Loeb, 1999; Sullivan & Sullivan, 2019). Johnson (2007) concluded that food and shelter are important determinants of habitat quality and may thus influence habitat selection of small mammal species in heterogeneous landscapes. In the absence of larger‐scale disturbances, habitat features and structural diversity in primeval forests might be relatively stable, while food availability is distinctly driven by the masting behavior of trees.

To our knowledge, habitat use of small mammals as a function of temporally varying seed rain has not been studied in primeval forest sites with differing disturbance history. To address this gap, we used a unique long‐term dataset of standardized live trapping of small mammals and records of seed rain to model the habitat use of the most common small mammal taxa in the largest remaining alpine beech‐dominated primeval forest in the Wilderness Area Dürrenstein, Lower Austria. Specifically, we assessed the effects of mast seeding on *Apodemus* spp. and *Myodes glareolus* densities across sites with differing disturbance history, including frequent small‐scale and rare medium‐scale natural disturbances on primeval or old‐growth forest patches, as well as managed forest without natural disturbances. To do so, we used hierarchical Bayesian N‐mixture models to estimate temporal changes in rodent abundance for each of the sites, as a function of seed rain. Because abiotic conditions are known to affect the detection probability of the target species (Wróbel & Bogdziewicz, 2015), we also considered the effects of time‐varying detection probability and weather (precipitation, temperature).

We hypothesized a site‐specific effect of seed rain on population densities of both small mammal taxa, expressed as a spillover in naturally formed gaps. As other studies detected an increase in *M. glareolus* into human‐altered open habitats when population density was high (Hansson, 1996; Sundell et al., 2012; Zwolak et al., 2018), we specifically expected a site‐specific response by *M. glareolus* between forest patches with medium‐scale natural disturbance events and reduced canopy cover versus sites with small‐scale disturbance events or no disturbance.

## 2 METHODS

### 2.1 Study area

The Wilderness Area Dürrenstein (WAD; 47°48′ to 47°45′N, 15°01′ to 15°07′E) is located within the northern Limestone Alps of Lower Austria, Austria. The climate of the region is submaritime with long winter periods and short cool summers. Annual precipitation (max. 2,300 mm) shows a bimodal pattern, reaching one maximum during the vegetation period and another one at wintertime. The protected area of the WAD covers 3,500 ha in total from which approximately 300 ha is declared as a strictly protected area (International Union for Conservation of Nature category Ia), the primeval forest Rothwald, which has never been logged (Kral & Mayer, 1968; Splechtna & Splechtna, 2016). The forests are classified as *Asperulo‐Abieti‐Fagetum* and as *Adenostylo‐glabrae‐Fagetum*, a higher altitude subtype of a *Galio‐odorati‐Fagetum* (Willner & Grabherr, 2007). European beech *Fagus sylvatica* dominates on all sites and particularly on the slopes, with Norway spruce (*Picea abies*) and silver fir (*Abies alba*) as the other common species. The disturbance history of the area is well documented, and the disturbance regime is characterized by frequently occurring low severity disturbances and less frequent medium‐scale disturbance events (Splechtna & Gratzer, 2005; Splechtna et al., 2005). The disturbance history shows strong temporal variation at centennial timescales (Splechtna et al., 2005).

**FIGURE 1:**
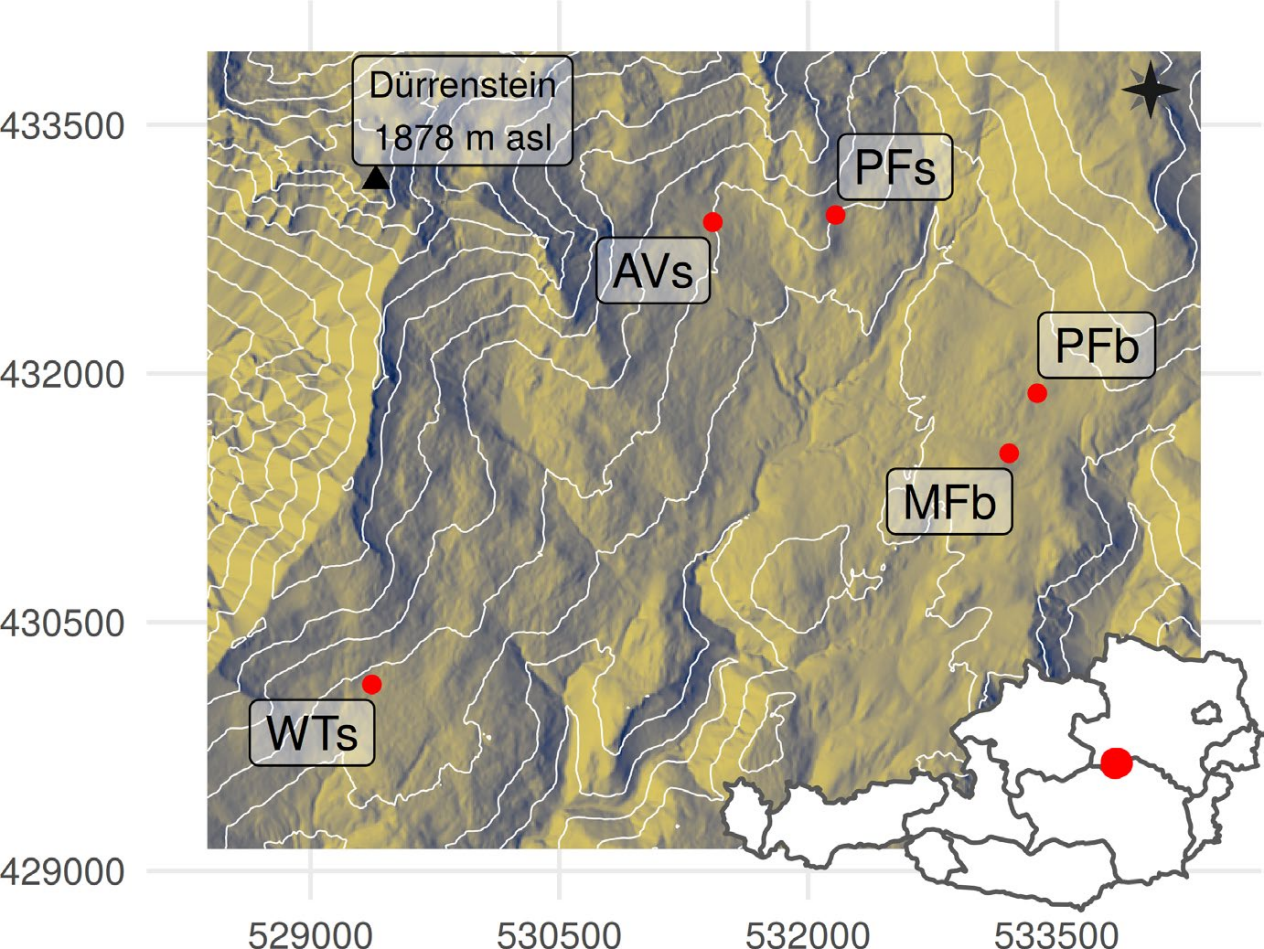
Location of the study sites for small mammal live trapping in the Wilderness Area Dürrenstein (WAD) in Austria. The location of the WAD itself is highlighted on the inset map of Austria (bottom right). The hillshade and contour lines (each line represents 100 m differences in altitude with a range between 800 and 1,800 m a.s.l.) visualize the two main geomorphological landforms: a flat basin in the eastern part of the WAD (primary forest site in the basin = PFb and managed forest site = MFb) and the southeastern slopes of the mountain Dürrenstein (primary forest site at the slopes = PFs, avalanche site = AVs, windthrow site = WTs). At AVs, an avalanche occurred in 2009, and at WTs, a windthrow hit the area in 1990. There was no recent natural disturbance event at the primeval forest site PFs or at the two sites in the basin. Data sources: digital elevation model: http://www.geoland.at (CC BY 4.0); administrative units of Austria: made with Natural Earth. Projected coordinate system: ETRS89/Austria Lambert (EPSG: 3416)

We established five study sites with differing disturbance history and/or geomorphology, with three sites situated at the southeastern slopes of the summit Dürrenstein (1,878 m a.s.l., 47°47′N, 15°04′E) and two sites located in a basin in ~4‐km linear distance to the summit Dürrenstein (Figure 1).

The study sites on the *slopes* represent the following three disturbance scenarios:

1. A primeval forest site with frequent small‐scale disturbance history (PFs) of approx. 240 ha; PFs is characterized by old individuals of *F. sylvatica*, regeneration is patchily distributed, and calcareous C‐horizon is partly exposed, offering many holes accessible for small animals.
2. A primeval forest site where an avalanche occurred in 2009, creating a medium‐scale disturbance patch (AVs) of 10.1 ha size with a length of over 1,000 m and a width of up to 120 m. *P. abies* established rapidly in the middle parts, while the avalanche runout zone is characterized by a high amount of coarse woody debris and gravel. Eleven years after the disturbance event, open grassland occupied around half of the site (Brenn, 2018).
3. A windthrow area (WTs) of 10 ha size (i.e., a medium‐scaled disturbance event), which was formed in 1990 in an old‐growth forest stand close to PFs. The area was not cleared from logs and provides a heterogeneous habitat with dense thickets intermixed with patches of grassland.

The study sites in the *basin* represented the following two disturbance scenarios:

1. A primeval forest site with frequent small‐scale disturbance history (PFb) of approx. 60 ha; it is characterized by a high amount of deadwood and densely mixed regeneration and a higher ground vegetation cover compared with PFb.
2. A managed forest site without occurrence of natural disturbances (MFb); MFb is dominated by *P. abies*, intermixed with *F. sylvatica* and *A. alba*. Ground vegetation is dominated by *Vaccinium myrtillus*, which is less common at all other sites. The managed forest (MFb) is adjacent to PFb and other beech‐dominated forest stands.

We monitored seed rain on the plots PFs and PFb, representing the slope and the basin, respectively. Small mammal live trapping was done on all five study sites (hereafter referred to as mammal sites; Table 1).

**TABLE 1:** Description of study sites for small mammal trapping

Abbreviation	Site description	Geomorphology	Period of records	Canopy cover	Disturbances, spatial scale	
PFb	Primeval forest	Basin	2004–2019	Closed	Small‐scale gaps	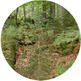
MFb	Managed forest	Basin	2004–2019	Closed	Forest management, logging	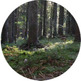
WTs	Uncleared windthrow (in 1990) on old‐growth forest site	Slope	2004–2019	Heterogeneous	Medium scale	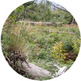
PFs	Primeval forest	Slope	2004–2019	Closed	Small‐scale gaps	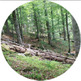
AVs	Avalanche (in 2009) on primeval forest site	Slope	2012–2019	Open	Medium scale	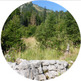

Note

All sites are located in the Wilderness Area Dürrenstein (47°48′ to 47°45′N, 15°01′ to 15°07′E). The sites PFs, PFb and AVs are part of the primeval forest Rothwald, which has never been logged and is categorized as a strict nature reserve (i.e., International Union for Conservation of Nature category Ia).

### 2.2 Mammal trapping

To estimate the abundance of small mammals, we conducted live trapping between 2004 and 2019. Trapping sessions were carried out between May and October with one to three sessions each year. The duration of a trapping session varied between 2 and 5 consecutive trap nights (see Appendix S1 for details about the timing and duration of the trapping sessions). Trapping grids on mammal sites were composed of 5 × 5 trap stations arranged on a grid with 15 m distance between stations. Accounting for the elongated shape of the avalanche site, we used a modified grid design there, placing 44 trap stations on three subgrids while maintaining trap distance. We placed two traps of different manufacturers (i.e., wooden box traps, Sherman traps, tube traps, and trip traps) at each trap station and covered them with vegetation or other organic material to prevent extreme temperatures. We baited each trap with butter cookies, peanut butter, and a piece of apple (Cody & Smallwood, 1996). Traps were set in the evening and checked each morning. Species or genera were identified according to Niethammer and Krapp (1978, 1982). Individuals of the genus *Apodemus* were not identified to species level, as it is not possible to reliably discriminate between different members of the subgenus *Sylvaemus* in Central Europe purely based on morphological metrics measured under field conditions (Barčiová & Macholán, 2009). Three different *Apodemus* species potentially occur within our study area: *A*. *flavicollis*, *A. sylvaticus*, and *A. alpicola*. Owing to the protection status of the research area, we could not use artificial permanent marking methods such as passive integrated transponder tags or metal tags that would otherwise accumulate in the forest. All fieldwork was conducted in accordance with the reserve administration and the scientific advisory board of the Wilderness Area Dürrenstein and permits by the Government of Lower Austria, Nature Conservation Division (RU5).

### 2.3 Seed rain

To capture temporal variation in seed rain, we took advantage of an ongoing long‐term study on seed production in two of our study plots (Gratzer, unpublished data). Seed rain was monitored at the plots PFs (representing the slope) and PFb (representing the basin) in 100 × 100 m plots using geostatistical grid designs with 81 seed traps in 2003 and from 2006 to 2018 (for details about the grid design, see Appendix S2). The traps consisted of plastic troughs with a basal area of 0.24 m^2^ covered with wire mesh to prevent further dispersal or predation of seeds. We emptied all seed traps in early spring right after snowmelt and additionally during late October/November unless unpredictable snow cover prevented us to enter the area. Collected seeds were separated from leaf litter and other organic material and counted for each tree species and seed trap. Seeds fallen between late summer and the following spring were summed up for each seed trap as annual estimates, and we used log‐transformed mean values of 81 seed traps and scaled the number of seeds to [seeds/m^2^] for further analysis. We combined the number of seeds of *P. abies* and *A. alba* (hereinafter referred to as conifer seeds), as their seed rain was positively correlated (Spearman's rho = 0.69, *p* = 4.5e−05, *n* = 28; see Appendix S3 for details about the correlation between seed rain of different tree species).

### 2.4 Microclimate

To account for microclimatic variation among plots and trap nights, which can influence the activity and capture numbers of *Apodemus* spp. and *M. glareolus* in a species‐specific manner (Wróbel & Bogdziewicz, 2015), we used a fine‐scale model (Kearney et al., 2020) to estimate hourly mean temperatures for each study site using the R‐package microclima (Maclean et al., 2019; see Appendix S4 for details about the parameters we set). To validate the estimated temperatures, we used data obtained from a weather station close to the mammal site WTs (see Appendix S4). Aggregated site‐specific mean values between 19:00 and 06:00 CET were used to account for different temperature conditions during a trap night. We further obtained estimates of daily precipitation using the function “microclimaforNMR” of the same R package (Kearney & Porter, 2017).

### 2.5 Statistical analysis

To obtain reliable abundance estimates of unmarked rodents while accounting for heterogeneity in detection probability and a small number of sampling plots, we developed N‐mixture models (Royle, 2004) for two rodent populations using a time‐for‐space substitution (Costa et al., 2019; Yamaura et al., 2011). Previous comparisons of this approach with mark–release–recapture (MRR) models for different small mammals revealed comparable results, and importantly, additional simulation results of Kellner et al. (2013) demonstrated that the N‐mixture approach showed less mean absolute bias of the model estimate from the true value than MRR models when detection probability was heterogeneous.

In live‐trapping studies, detection probability can be used interchangeably with capture probability, as it is only possible to detect an individual when captured. However, the trappability of rodents is unlikely to be static and it is important to consider potential factors that might influence detection probability (Kéry & Schaub, 2012). Hierarchical modeling approaches are ideally suited for this task as detection probability and population size can be linked in a hierarchical manner and estimated simultaneously. We therefore jointly analyzed potential trap habituation and weather effects on detection probability for both rodent taxa using a hierarchical modeling approach.

To implement the model, we summarized live‐trapping data for each rodent taxon as the number of captured individuals $C_{\mathrm{ij}}$ within a trap night j at session i. According to the AnAge database, gestation takes about 23 days for *A. sylvaticus*, 26 days for *A. flavicollis*, and 20 days for *M. glareolus* (Magalhães & Costa, 2009; Tacutu et al., 2018). Different sessions at a site had a minimum time lag of 35 days, and we assumed those primary periods to be open to population changes. We assumed demographic closure within secondary periods (up to five consecutive trap nights) and equal detection probability for all individuals of a taxon within a trap night. In summary, we used 115 sessions of 5 different sites as if they were spatial replications, capturing 455 nights of trapping.

We fit the models using a Markov chain Monte Carlo (MCMC) algorithm (Just Another Gibbs Sampler, JAGS version 4.3; Plummer, 2003) for both rodent taxa separately. We used noninformative priors and standardized all continuous covariates to facilitate convergence. JAGS was called from within R 3.6.3 (R Core Team, 2020) using the R‐package jagsUI (Kellner, 2019). We set the MCMC algorithm to run on three chains using random initial values with 800,000 iterations, a burn‐in and adaptation period of 100,000 draws, and a thin rate of 200 yielding 10,500 total samples. Convergence was assessed visually via trace plots, and we ensured that all $\hat{R}$‐values were below 1.1 (Brooks & Gelman, 1998).

The abundance of each rodent taxon at session i was modeled using a Poisson lognormal binomial mixture model (Kéry & Schaub, 2012):

$$N_{i}∼\mathrm{Poisson}\left(\lambda_{i}*A_{i}\right)$$

$$\log\left(\lambda_{i}\right)=\log\left(A_{i}\right)+\beta_{0}+\beta_{1}*{\mathrm{Site}}_{i}+\beta_{2}*{\text{seedrain beech}}_{i}+\beta_{3}*{\text{seedrain conifers}}_{i}+\beta_{4}*{\mathrm{Julian}}_{i}+\beta_{5}*{\mathrm{Julian}}_{i}^{2}+\beta_{6}*{\text{seedrain beech}}_{i}*{\mathrm{Site}}_{i}+\epsilon_{i}$$

$$\epsilon_{i}∼\mathrm{Normal}\left(0,\;\tau \right)$$

where $\lambda_{i}$ represents the mean abundance per hectare during session i. $\beta_{0}$ represents the intercept, whereas $\beta_{1}$ to $\beta_{6}$ are slope coefficients for study site, seed rain of *F. sylvatica*, seed rain of conifers, Julian day, a quadratic term of Julian day, and an interaction term between seed rain of *F. sylvatica* and mammal site, respectively. We added this interaction as we only measured seed rain on study sites, where all three main tree species (*F. sylvatica*, *A. alba*, and *P. abies*) had reached the reproductive age (i.e., at PFb and PFs) and seed rain of *F. sylvatica* could potentially occur due to the presence of parent trees. In contrast, the interaction between seed rain of conifers and mammal sites was not included as dispersal distances of conifer seeds are considerably larger compared with *F. sylvatica* (Kutter, 2007), potentially allowing for seed rain even on sites without occurrence of parent trees in situ. Therefore, the spatial distribution of conifer seeds was assumed to be more homogenous between forest gaps and the surrounding forests and site‐specific effects should be of minor importance. As the trapping grid at the avalanche site AVs was larger compared with the other mammal sites, we included an offset $A_{i}$ to account for differences in the areal extent between mammal sites. Therefore, $\lambda_{i}$ can be interpreted as density [individuals*ha^‐1^]. Finally, we added a random effect $\epsilon_{i}$ to account for extra‐Poisson variation in the latent abundance (i.e., overdispersion).

We specified the model for the detection process as follows:

$$C_{\mathrm{ij}}|N_{i}∼\mathrm{Binomial}\left(N_{i},p_{\mathrm{ij}}\right)$$

$$\begin{array}{c} \text{logit}\left(p_{\mathrm{ij}}\right)=\alpha_{0}+\alpha_{1}*{\text{Night of Session}}_{\mathrm{ij}}+\alpha_{2}*{\text{Temperature}}_{\mathrm{ij}}+\alpha_{3}*{\text{Temperature}}_{\mathrm{ij}}^{2}\\ +\alpha_{4}*{\text{Precipitation}}_{\mathrm{ij}}+\alpha_{5}*{\text{Temperature}}_{\mathrm{ij}}*{\text{Precipitation}}_{\mathrm{ij}} \end{array}$$

where $p_{\mathrm{ij}}$ is detection probability at session i and trap night j. $\alpha_{0}$ represents the intercept, and $\alpha_{1}$ to $\alpha_{5}$ are slope coefficients for the night of the session, the mean temperature between 19:00 and 06:00 CET of the preceding night, a quadratic term of the mean temperature, the sum of precipitation of the previous day, and an interaction between precipitation and temperature, respectively. We added a quadratic term of the mean temperature as we expected extreme temperatures (very hot and very cold) to have a negative effect on the detection probability because small mammals might adapt foraging behavior according to their thermal neutral zone (Juliana & Mitchell, 2016). As relative humidity increases with precipitation and both humidity and temperature affect thermoregulation of mammals (Bronson & Perrigo, 1987), we included an interaction term between ambient temperature and precipitation to transcribe apparent temperature.

Furthermore, weather conditions influence the hunting activity of predators, such as *Mustela nivalis* (Brandt & Lambin, 2005), which in turn might affect rodent activity to avoid predation (Vickery & Bider, 1981).

## 3 RESULTS

### 3.1 Seed rain

**FIGURE 2:**
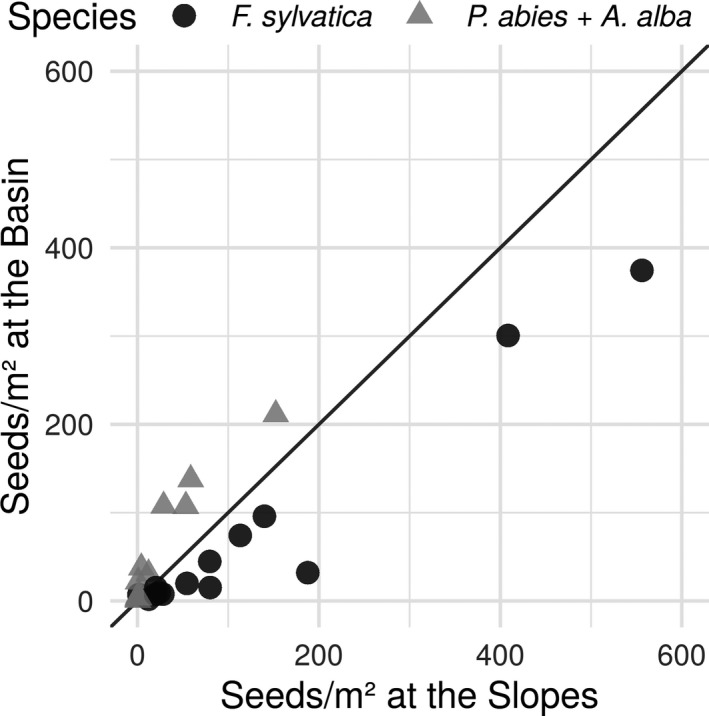
Arithmetic mean values of seeds/m^2^ for *Fagus sylvatica* (black points) and conifer species (gray triangles). Each point represents paired mean values for each masting period sampled from 81 seed traps at two geomorphological landforms (basin and slope). The straight line (intercept = 0 and slope = 1) indicates a hypothetical perfect correlation (i.e., not derived from data). Points below the straight line indicate more seeds at the slopes; points above the straight line indicate relatively more seeds at the basin

Seed production of the three dominant tree species varied strongly between study years, with overall mean values of *F. sylvatica* ranging from less than 4.3 seeds per m^2^ in 2006 to 465.3 seeds per m^2^ in 2011 (Figure 2). Conifer seeds were most abundant in 2003 and scarcest in 2014, with overall mean values of 181.8 and 0.8 seeds per m^2^, respectively (Figure 2). Seed rain of conifers was generally higher on the basin plot, whereas seed rain of *F. sylvatica* was more pronounced at the slopes (see Figure 2; for further details on the correlation of seed rain between geomorphological landforms basin/slope and tree species see Appendix S3). However, mean values of seed rain per m^2^ between the basin and the slopes were highly correlated (Spearman's rho*_F. sylvatica_* = 0.93, Spearman's rho*_P. abies_*_+_ *_A. alba_* = 0.94). The number of conifer seeds was higher than average in 2003, 2011, 2015, and 2018 both on the slope and in the basin. Masting of *F. sylvatica* was above average in 2003, 2007 (basin plot only), 2011, 2014, and 2016 (slope plot only).

### 3.2 Microclimate

Nightly estimates of mean temperature differed only slightly among sites. For example, the primeval forest at the slope PFs was on average 0.35°C ± 0.17 colder than PFb, and 0.26°C ± 0.18 colder than AV. Furthermore, we found that WTs mean nightly temperatures during the summer months were highly correlated with data from the nearby weather station (Appendix S4) supporting the validity of our estimates.

### 3.3 Small mammal captures

**FIGURE 3:**
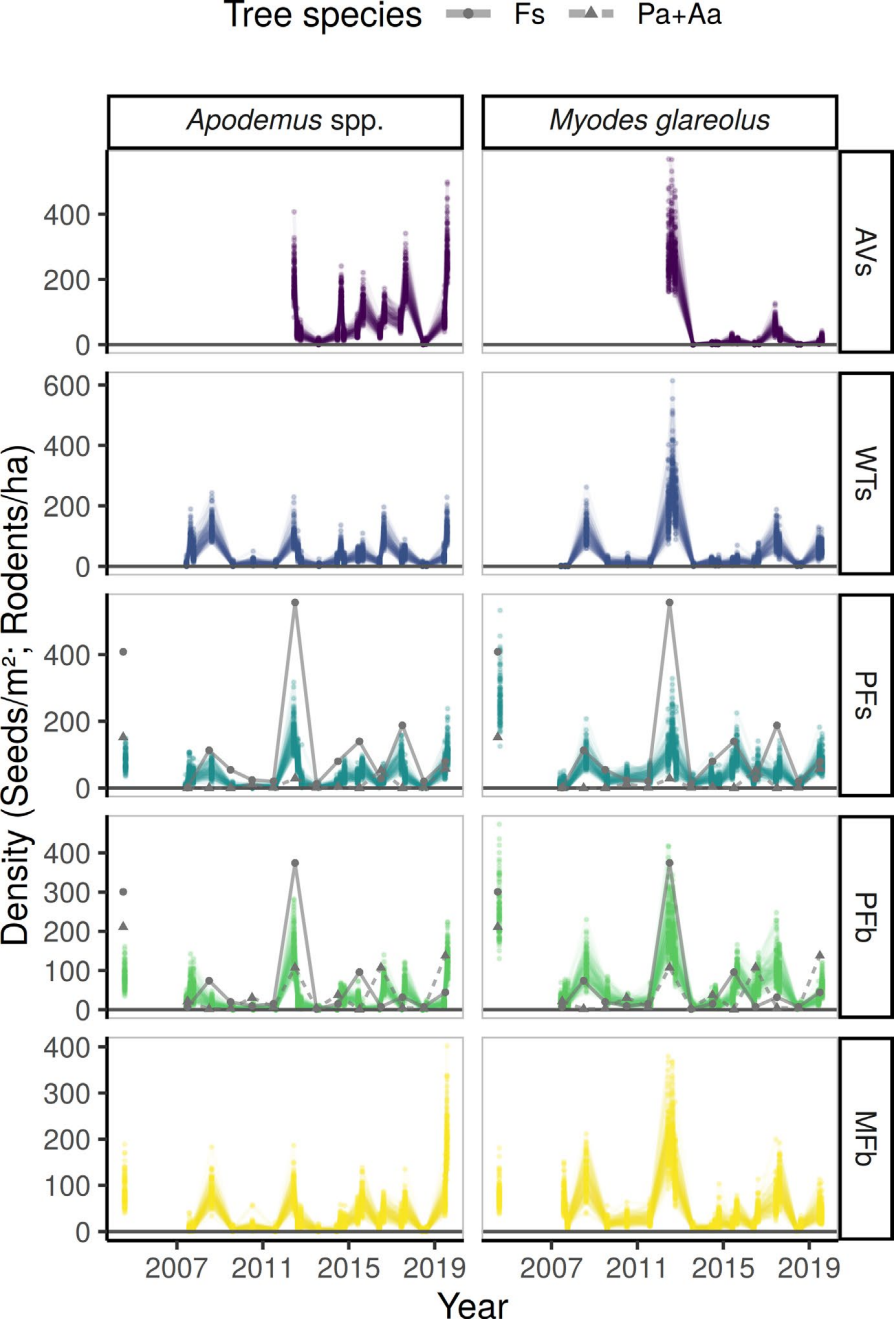
Temporal patterns of estimated rodent density [individuals per ha] and measured seed rain of the dominant tree species [seeds per m²]. Gray lines indicate arithmetic mean values of seeds per m² of *Fagus sylvatica* (Fs) and conifers (Pa+Aa: sum of *Picea abies* and *Abies alba*). Rodent trapping was conducted at sites PFb = primary forest in the basin, MFb = managed forest in the basin, PFs = primary forest at the slopes, WTs = windthrow at the slopes, and AVs = avalanche at the slopes. Seed rain of Fs and conifer species was sampled via 81 seed traps in the basin (PFb) and at the slopes (PFs), where all three main tree species (*F. sylvatica*, *A. alba*, and *P. abies*) had reached the reproductive age. Colored points depict a random sample of 100 posterior predictions of rodent density for each site and trapping session (lines connect estimates to support visual inspection). Please note: Date for seed rain was fixed at 1 July of the year following seed production (e.g., 01.07.2007 represents the seed rain of the period between autumn 2006 and the subsequent winter in 2007)

In 455 trap nights, we recorded 2,385 *Apodemus* spp. and 2,195 *M*. *glareolus* captures. Capture numbers were highly variable between years for both taxa (see Figure 3 for population density estimation). In addition to the two target taxa, we frequently trapped *Glis glis* (*n* = 281), *Muscardinus avellanarius* (*n* = 37), *Sorex araneus* (*n* = 79), *Sorex minutus* (*n* = 81), *Sorex alpinus* (*n* = 69), and *Sorex* sp. (i.e., not determined to species level; *n* = 30). Furthermore, we recorded rare captures of *Microtus agrestis* (*n* = 11), *Microtus subterraneus* (*n* = 12), and *Crocidura suaveolens* (*n* = 1). *Mustela* sp. and *Vipera berus* occurred at WTs and AVs only, where the latter was seen coincidentally and did not enter the traps.

### 3.4 Model performance

Both N‐mixture models showed only slight overdispersion with $\hat{C}$‐values of 1.08 in *Apodemus* spp. and 1.09 in *M. glareolus*. Bayesian *p*‐values based on a chi‐squared discrepancy measure were .18 for *Apodemus* spp. and .15 for *M. glareolus*, indicating acceptable fit of the models to the data.

### 3.5 Drivers of detection probabilities

Detection probabilities were generally affected by abiotic conditions (Table 2). For *Apodemus* spp., detection probability increased with increasing precipitation and over consecutive nights within a session (see Figure S5.1), while the detection probability of *M. glareolus* was affected by the interaction between temperature and precipitation and the squared term of the mean nightly temperature (Figure S5.2).

**TABLE 2:** Drivers of rodent density and detectability of *Apodemus* spp. and *Myodes glareolus*

		*Apodemus* spp.		*Myodes glareolus*	
Submodel	Covariate	Posterior mean	Posterior *SD*	Posterior mean	Posterior *SD*
λ	intercept	2.89*	0.39	3.73*	0.26
	*Fagus sylvatica* seeds	0.7*	0.25	0.85*	0.13
	Conifer seeds	0.31*	0.15	0.27*	0.09
	Julian	0.16	0.14	0.09	0.09
	Julian²	−0.1	0.13	−0.22*	0.08
	SiteMFb	0	0.00	0	0.00
	SitePFs	0.21	0.43	0.14	0.22
	SitePFb	−0.15	0.41	0.09	0.19
	SiteAVs	0.68	0.53	−2.85*	0.46
	SiteWTs	0.33	0.49	−0.93*	0.29
	Site:*Fagus sylvatica* seeds	−0.07	1.02	−0.73*	0.09
p	intercept	−1.52*	0.23	−1.08*	0.29
	Precip:Temp	−0.04	0.05	0.12*	0.06
	Night of Session	0.23*	0.03	0.03	0.02
	Temp	−0.09	0.06	−0.09	0.06
	Temp²	−0.02	0.05	−0.1*	0.04
	Precip	0.1*	0.04	−0.06	0.05

Note

Posterior mean and standard deviation of our N‐mixture models for *Apodemus* spp. and *Myodes glareolus*. Covariates of the submodel λ were included in the abundance process, while covariates of p describe the detection process.

* Parameter has a 95% credible interval that does not include 0

### 3.6 Drivers of rodent density

**FIGURE 4:**
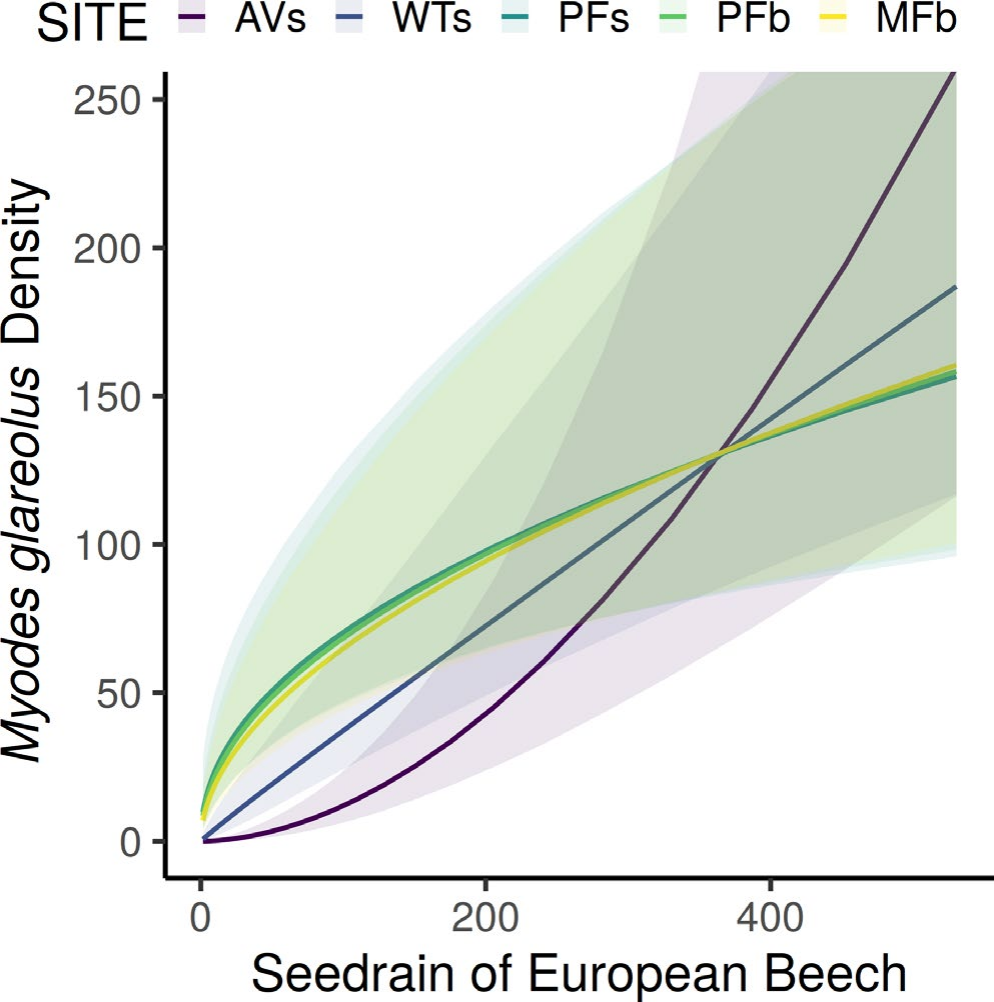
Marginal effect of seed rain [seeds*m^−2^] of *Fagus sylvatica* on the density estimation of our N‐mixture model for *Myodes glareolus* for each study site. The 95% credible interval of the posterior distribution does not include zero for the interaction between study site and seed rain of *Fagus sylvatica*. Other covariates are held constant at their mean value. Standardized seed rain was back‐transformed to facilitate interpretability

Estimated population densities were highly variable between sites and years for both species (Figure 3). The N‐mixture models indicated that seed rain of *F. sylvatica* and conifers had a positive effect on population density of both rodent taxa (Table 2). Additionally, the relationship between seed rain of *F. sylvatica* and the density of *M. glareolus* was site‐specific as indicated by the interaction term with site ID (Figure 4). In years following moderate or low seed crops, *M. glareolus* was less abundant at sites with medium‐scale disturbances. Density of *M. glareolus* generally peaked during the summer months and was lower during spring and autumn, as the squared term of Julian day did not include zero.

## 4 DISCUSSION

In our study, seed rain of the dominant tree species was a strong driver for the density of *M. glareolus*, but its effect differed between study sites. When seed rain of *F. sylvatica* was low, *M. glareolus* inhabited almost exclusively forest habitats with canopy cover, including both primeval forest stands with frequent small‐scale disturbances (PFs and PFb) and the managed forest stand without natural disturbances (MF). However, in years with high overall density following bumper crops of seeds, the species even occupied open sites (created by medium‐scale natural disturbances) in large numbers, exceeding local densities in their commonly preferred habitat. Corresponding to our hypothesis, the observation of spillover effects in *M. glareolus* applies both for the avalanche patch on the primeval forest site (AVs) and for the windthrow patch (WTs) on a formerly old‐growth forest site. However, our results do not indicate site‐specific responses to seed rain for *Apodemus* spp.. These main findings are in line with other studies on *M. glareolus* on forest sites with adjacent open habitats, although the open patches in these studies were not created by natural disturbances but stemmed from human intervention (i.e., clear‐cuts, fields, and meadows; Hansson, 1996; Sundell et al., 2012; Zwolak et al., 2018). Such changes in relative abundance of *M. glareolus* in different habitats in the course of differing overall population densities might be the reason why the species has been described as both a forest specialist (Torre & Arrizabalaga, 2008) and a habitat generalist (Gliwicz & Glowacka, 2000).

In contrast, we did not find different densities of *Apodemus* spp. or *M. glareolus* between primeval forest sites with frequent small‐scale disturbances and canopy cover and adjacent managed forest without occurrence of natural disturbances. These findings are in line with results from Zwolak et al. (2016), who compared abundances of *A. flavicollis* and *M. glareolus* between managed shelterwood and closed‐canopy beech stands without observing distinct differences. However, Gasperini et al. (2016) found strong effects of silvicultural management practices (coppicing and conifer afforestation) on the population density of *A. flavicollis*, *A. sylvaticus* and *M. glareolus* with positive effects of coppicing on all three species, and negative effects of conifer plantations on *A. flavicollis* and *M. glareolus*. Carey and Johnson (1995) showed that small mammal communities of the Pacific Northwest were similar in composition between naturally regenerated young forests and clear‐cutting regenerated (managed) young forests compared with old‐growth forests, but their density was 1.5 times higher within the old‐growth forests. Primeval forests in alpine landscapes are typically characterized by a mosaic of different forest successional stages as a consequence of stand‐replacing disturbances intermixed with different forest development stages caused by finer scaled disturbances such as forest gaps. The occurring pattern of various transitional stages in fully natural primeval forest ecosystems may create a spatiotemporal dynamic source–sink situation for *M. glareolus*.

The intermediate effect size of seed rain of *F. sylvatica* on the density of *M. glareolus* in the windthrow area meets the expectation of the species´ core habitat being generally associated with advanced forest successional stages in Central Europe (Ecke et al., 2002). The progression of canopy cover along with a pronounced amount of structural habitat elements, such as root plates and logs in consequence of a windthrow event that occurred in 1990, probably enhances habitat quality for *M. glareolus*. In contrast, the avalanche site was disturbed in 2009 and still showed clear forest gap characteristics in 2019, almost lacking canopy cover. However, the site is rich in structural ground elements, such as logs, boulders, herbaceous vegetation, and tall grasses. As both sites of natural disturbance events mainly differ in terms of canopy cover, we suggest that this is a crucial factor determining habitat quality for *M. glareolus*.

Annually fluctuating resource dynamics, such as mast seeding, might distinctly drive overall abundance and habitat selection of primary consumers such as granivorous rodents and birds (Bogdziewicz et al., 2016). However, it is unclear how natural disturbance dynamics and masting cycles of forest trees interact as driving factors of population dynamics of granivorous animal populations. This important knowledge gap, concerning the interaction between factors that determine the height of the density peak, was recently highlighted in a review of population cycles and outbreaks of small rodents (Andreassen et al., 2020). In this study, we focused on dominant granivorous ground‐dwelling rodent taxa *Apodemus* spp. and *M. glareolus*, with the former being a seed specialist (Selva et al., 2012) and the latter feeding on a broader spectrum of different food sources (Abt & Bock, 1998; Čermák & Ježek, 2005). Although *M. glareolus* switches its diet and consumes more seeds after mast events (Selva et al., 2012), the proportion of nongranivorous food items is still higher compared with *A. flavicollis* resulting in effectively higher food abundance, especially in areas rich in alternative food resources.

Along with a decrease in canopy cover, other habitat characteristics also change after natural disturbances, including the amount and spatial distribution of seed rain, the cover and net biomass of herbaceous ground vegetation and leaf litter, the degree of insolation, temperature amplitudes, and therefore microclimate (Abd Latif & Blackburn, 2010; Canham et al., 1990; Clinton, 2003). Three different species of the genus *Apodemus* were likely to occur in our study area: *A*. *flavicollis* is commonly described as a forest specialist, avoiding open habitats but showing a preference for forest edges, while *A. sylvaticus* is described as a habitat generalist frequently occurring in open landscapes (Schlinkert et al., 2016). Preferred habitats of *A. alpicola* typically include grassy areas intermixed with boulders in mountainous forest regions (Spitzenberger & Englisch, 1996). A meta‐analysis by Bogdziewicz and Zwolak (2014) found a higher relative abundance index for *A. sylvaticus* and for *A. flavicollis* in clear‐cuts compared with unharvested temperate forests. Furthermore, population density of both species has been positively related to coppicing activities (Gasperini et al., 2016). However, our results did not indicate differences in density between disturbance sites and the forest sites with canopy. As we could not identify *Apodemus* specimens to species level due to well‐known constraints of morphological traits under field conditions (Barčiová & Macholán, 2009; Reutter et al., 1999), species composition of the subgenus *Sylvaemus* might have differed between our study sites, which might have masked differing species‐specific local densities. Nonetheless, according to our model, seed rain was the only covariate showing a clear effect on the density of *Apodemus* spp., which underlines the strong bottom‐up influence of seed rain on overall population density of seed specialists.

N‐mixture models explicitly combine the abundance process and the detection process in a unified framework. The estimation of detection probability is crucial, as the assumption of equal detection probability is very likely to be violated in most small mammal studies, which is a common criticism of population indices as a proxy for abundance (McKelvey & Pearson, 2001; Slade & Blair, 2000). In case of *Apodemus* spp., our model suggests a positive influence of the night of the session on detection probability and it could be concluded that habituation affects the trappability of this genus. Some studies circumvent this problem by placing the traps a few days before the actual trapping sessions without setting them up (Flowerdew et al., 2017; Kellner et al., 2013), but there is neither a consensus about the duration nor is it conventional to make use of this option. Additionally, prebaiting may attract individuals from neighboring areas, which might introduce bias for the estimation of abundance (Barnett & Dutton, 1995). However, our model did not include information about individual identities and we cannot draw conclusions about individual heterogeneity of the habituation process. A possible impact of weather on the trappability of rodents has been known for decades, and different reasons have been suggested, among them niche separation (Drickamer & Capone, 1977) and predator avoidance (Vickery & Bider, 1981). For example, rainy conditions decrease activity in *Mustela nivalis* and *M. glareolus* (Brandt & Lambin, 2005), but have the opposite effect on *A. flavicollis* (Wróbel & Bogdziewicz, 2015). Our submodel for detection probability suggests an interaction between precipitation and temperature to be relevant for the trappability of *M. glareolus*. According to our model, an optimum temperature exists, where the negative impact of relatively high temperatures during the night is mitigated by the amount of rainfall of the preceding day. Although this interaction is fairly reasonable, we emphasize that further studies are needed to evaluate the interaction between different weather variables on detection probability of any species under study.

Combined together, our results confirm that forest stands act as source habitat characterized by relatively stable populations of *M. glareolus*, while more open areas act as sinks and become colonized only in times of overall high population numbers (Horne, 1983). It has been suggested that colonizers of habitat sinks are mainly composed of subdominant juveniles resulting in a higher carrying capacity due to lower social suppression between those unestablished immigrants (Horne, 1983), and Hansson (1996) confirmed a disproportional increase in density on clear‐cuts as well. Indeed, the highest density was found at AVs and the interaction term between seed rain and study site in the abundance submodel supports a disproportional increase in density at the avalanche site (AVs) after full mast events. As we did not have detailed data on demography, we cannot prove differences in age structure of *M. glareolus* populations between habitats. In addition to intraspecific competition, other studies pointed out that predation (Sundell et al., 2012), as well as interspecific competition with other species such as *Apodemus* spp., might cause habitat shifts in *M. glareolus* (Fasola & Canova, 2000; Zwolak et al., 2016).

Temporary high densities of *M. glareolus* within forest gaps might have serious implications for gap regeneration dynamics, as voles are known to have negative direct effects on tree seedling survival near forest edges (Ostfeld et al., 1997), especially when population densities are high (Ostfeld & Canham, 1993). Furthermore, *M. glareolus* is known to selectively feed on different tree species including *F. sylvatica* seedlings (Pigott, 1985). Extraordinary high local densities after mast events of *F. sylvatica*, as observed in 2012, therefore potentially affect tree species composition and impede forest gap regeneration.

The interplay between forest openings and frequently occurring mast events has species‐specific consequences for local rodent density. Natural abiotic disturbances are important drivers of forest dynamics in primeval forests (Splechtna et al., 2005; Thom & Seidl, 2016), and it is relevant to study the consequences and long‐term effects of gap formation on local population dynamics of animal taxa, such as granivorous and herbivorous rodent species. Following years of moderate or low seed crop, *M. glareolus* avoids open habitat patches within forests but colonizes those habitats in large numbers after full mast events. Therefore, the change in local density of *M. glareolus* among years is much more pronounced in forest gaps compared to sites with canopy cover. While other studies discovered this effect in human‐altered ecosystems (Hansson, 1996; Sundell et al., 2012; Zwolak et al., 2018), our study confirmed the interaction in a primeval forest, where gaps have been created by natural disturbance events.

## CONFLICT OF INTEREST

None declared.

## AUTHOR CONTRIBUTIONS

**Frederik Sachser:** Conceptualization (equal); data curation (equal); formal analysis (lead); investigation (equal); methodology (lead); project administration (equal); software (lead); supervision (supporting); validation (lead); visualization (lead); writing–original draft (lead); writing–review and editing (equal). **Mario Pesendorfer:** Conceptualization (supporting); data curation (supporting); formal analysis (supporting); investigation (supporting); methodology (supporting); supervision (equal); validation (supporting); visualization (supporting); writing–original draft (supporting); writing–review and editing (equal). **Georg Gratzer:** Conceptualization (lead); data curation (equal); funding acquisition (lead); investigation (lead); project administration (lead); resources (lead); supervision (equal); validation (equal); writing–original draft (supporting); writing–review and editing (equal). **Ursula Nopp‐Mayr:** Conceptualization (lead); data curation (equal); funding acquisition (equal); investigation (lead); project administration (equal); resources (lead); supervision (equal); validation (equal); writing–original draft (supporting); writing–review and editing (equal).

## Supporting information

Supplementary Material

## Data Availability

Data are deposited in the figshare repository: Sachser, Frederik; Pesendorfer, Mario; Gratzer, Georg; Nopp‐Mayr, Ursula (2021): Data for "Differential spatial responses of rodents to masting on forest sites with differing disturbance history". figshare. Dataset. https://doi.org/10.6084/m9.figshare.13860167.
