# Supplementary material for: Differential spatial responses of rodents to masting on forest sites with differing disturbance history
Source: Ecol Evol. 2021 Aug 13;11(17):11890–902. doi: 10.1002/ece3.7955 (PMC8427614; doi:10.1002/ece3.7955)
Supplement: Supplementary file 1 — Supplementary Material [file ECE3-11-11890-s001.pdf]

Differential spatial responses of rodents to masting on forest sites with differing disturbance history

Supplementary Material

Authors

Frederik Sachser[1,2], Mario Pesendorfer[1], Georg Gratzer[1], Ursula Nopp-Mayr[2]

[1] University of Natural Resources and Life Sciences, Vienna, Department of Forest- and Soil Sciences, Institute of Forest Ecology, Peter Jordan-Straße 82, 1190 Vienna, Austria

[2] University of Natural Resources and Life Sciences, Vienna, Department of Integrative Biology and Biodiversity Research, Institute of Wildlife Biology and Game Management, Gregor-Mendel-Straße 33, 1180 Vienna, Austria

ORCIDs

Frederik Sachser: 0000-0002-0124-909X
Mario Pesendorfer: 0000-0002-7994-7090
Ursula Nopp-Mayr: 0000-0002-0550-1096
Georg Gratzer: 0000-0002-6355-6562

Correspondence

Frederik Sachser
Email: frederik.sachser@boku.ac.at

Appendix S1: Trapping sessions of small mammal live captures

We conducted live trapping of small mammals in the years 2004 and 2007-2019. Trapping effort differed between sites and years (see Table S1.1).

Table S1.1: Information about timing (year and month) and duration (in trap nights) of the trapping sessions per site (MFb = managed forest, PFb = primary forest in the basin, PFb = primary forest at the slopes, WTs = windthrow site, AVs = avalanche site).

Year	Month	Duration	Sites
2004	7	2	MFb
2004	7	3	PFb
2004	8	3	PFs
2007	5	3	PFs
2007	6	2	WTs
2007	6	3	PFb
2007	7	3	MFb, PFs
2007	8	2	PFb
2007	8	3	WTs
2007	10	3	MFb, PFs, PFb, WTs
2008	8	3	MFb, PFs, PFb, WTs
2009	8	3	MFb, PFs, PFb, WTs
2010	7	3	MFb, PFs, PFb, WTs
2011	8	3	MFb, PFs, PFb, WTs
2012	6	2	AVs, WTs
2012	6	3	MFb, PFs, PFb
2012	8	3	MFb, PFs, PFb, AVs, WTs
2012	10	3	MFb, PFs, PFb, AVs, WTs

Year	Month	Duration	Sites
2013	8	3	MFb, PFs, PFb, AVs, WTs
2014	6	5	MFb, PFs, PFb, AVs, WTs
2014	8	5	MFb, PFs, PFb, AVs, WTs
2014	10	3	MFb, PFs
2014	10	5	PFb, AVs, WTs
2015	5	3	AVs
2015	6	5	MFb, PFs, PFb, WTs
2015	8	5	MFb, PFs, PFb, WTs
2015	9	3	AVs
2016	6	3	AVs
2016	6	5	MFb, PFs, PFb, WTs
2016	8	5	MFb, PFs, PFb, AVs, WTs
2017	5	5	AVs
2017	6	5	MFb, PFs, PFb, WTs
2017	8	4	AVs
2017	8	5	MFb, PFs, PFb, WTs
2018	6	2	WTs
2018	6	5	MFb, PFs, PFb, AVs
2018	8	5	MFb, PFs, PFb, AVs, WTs
2019	6	4	WTs
2019	6	5	MFb, PFs, PFb, AVs
2019	7	5	MFb, WTs
2019	8	5	PFs, PFb, AVs

Appendix S2: Location of seed traps on geostatistical grids

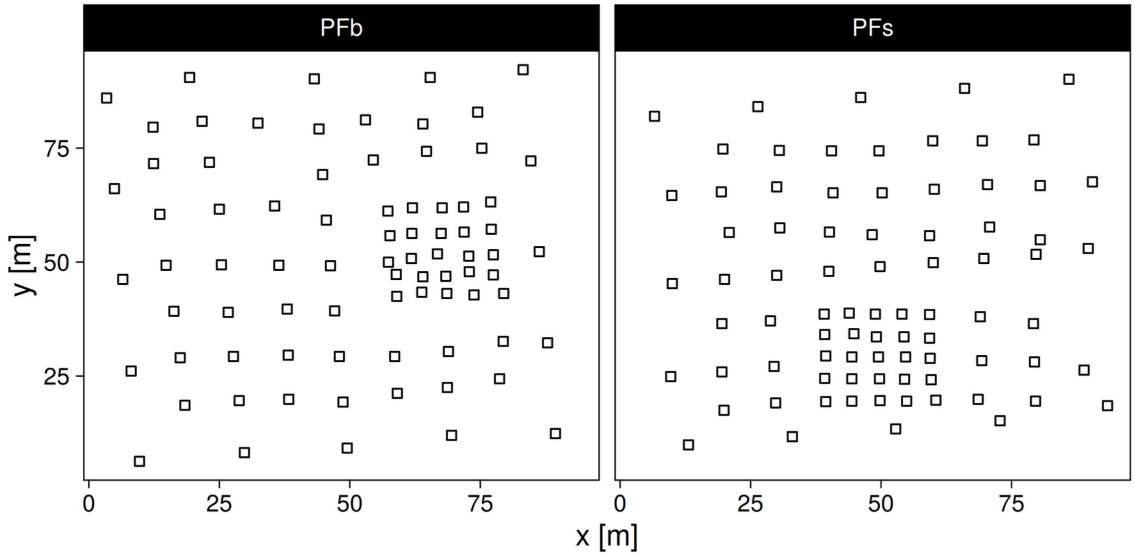

Figure S2.1: Geostatistical grid design for 81 seed traps at the study sites PFb (primary forest at the basin) and PFs (primary forest at the slopes). X and Y values describe the relative location of each trap within the grid.

Appendix S3: Seed rain of dominant tree species

Seed rain of the dominant tree species (*Fagus sylvatica*, *Picea abies*, *Abies alba*) varied between years and geomorphological landforms. *F. sylvatica* dominates particularly on the slopes and admixture of conifers (*P. abies*, *A. alba*) is more pronounced in the basin. Accordingly, conifer seeds are generally more frequent in the basin, while beechnuts are more frequent at the slopes (Figure S3.1). However, seed rain between geomorphological landforms are positively correlated for each dominating tree species:

- $F.\ sylvatica_{\text{Basin}}$ – $F.\ sylvatica_{\text{Slopes}}$: Spearman rho = 0.93, p = 1.0e-06, n = 14
- $P.\ abies_{\text{Basin}}$ – $P.\ abies_{\text{Slopes}}$: Spearman rho = 0.9, p = 1.4e-05, n = 14
- $A.\ alba_{\text{Basin}}$ – $A.\ alba_{\text{Slopes}}$: Spearman rho = 0.74, p = 2.4e-03, n = 14
- $P.\ abies_{\text{Basin}} + A.\ alba_{\text{Basin}}$ – $P.\ abies_{\text{Slopes}} + A.\ alba_{\text{Slopes}}$: Spearman rho = 0.92, p = 4.1e-06, n = 14

Seed rain of both conifer species are positively correlated (Spearman rho = 0.69, p = 4.5e-05, n = 28, Figure S3.2) and we used the sum of conifer seeds as a covariate for further modeling. There was no significant correlation between the amount of seeds of *F. sylvatica* and conifer species (neither for single species, nor aggregated values):

- *F. sylvatica* – *P. abies*: Spearman rho = 0.18, p = 3.7e-01, n = 28
- *F. sylvatica* – *A. alba*: Spearman rho = 0.09, p = 6.5e-01, n = 28
- *F. sylvatica* – *P. abies* + *A. alba*: Spearman rho = 0.11, p = 5.7e-01, n = 28

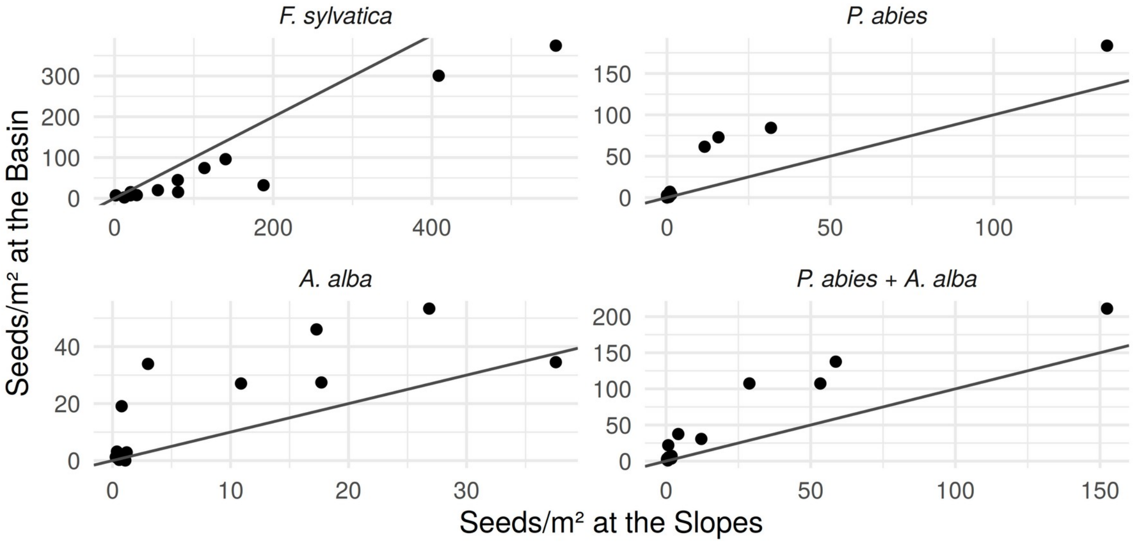

Figure S3.1: Seed rain of the dominant tree species *Fagus sylvatica*, *Picea abies*, *Abies alba* and additionally the sum of seeds per m² for conifer species (*P. abies* and *A. alba*) at both geomorphological landforms (basin and mountain slopes) within our study area. Straight lines depict a theoretical equal amount of seeds at both landforms (intercept = 0, slope = 1). Scales are free.

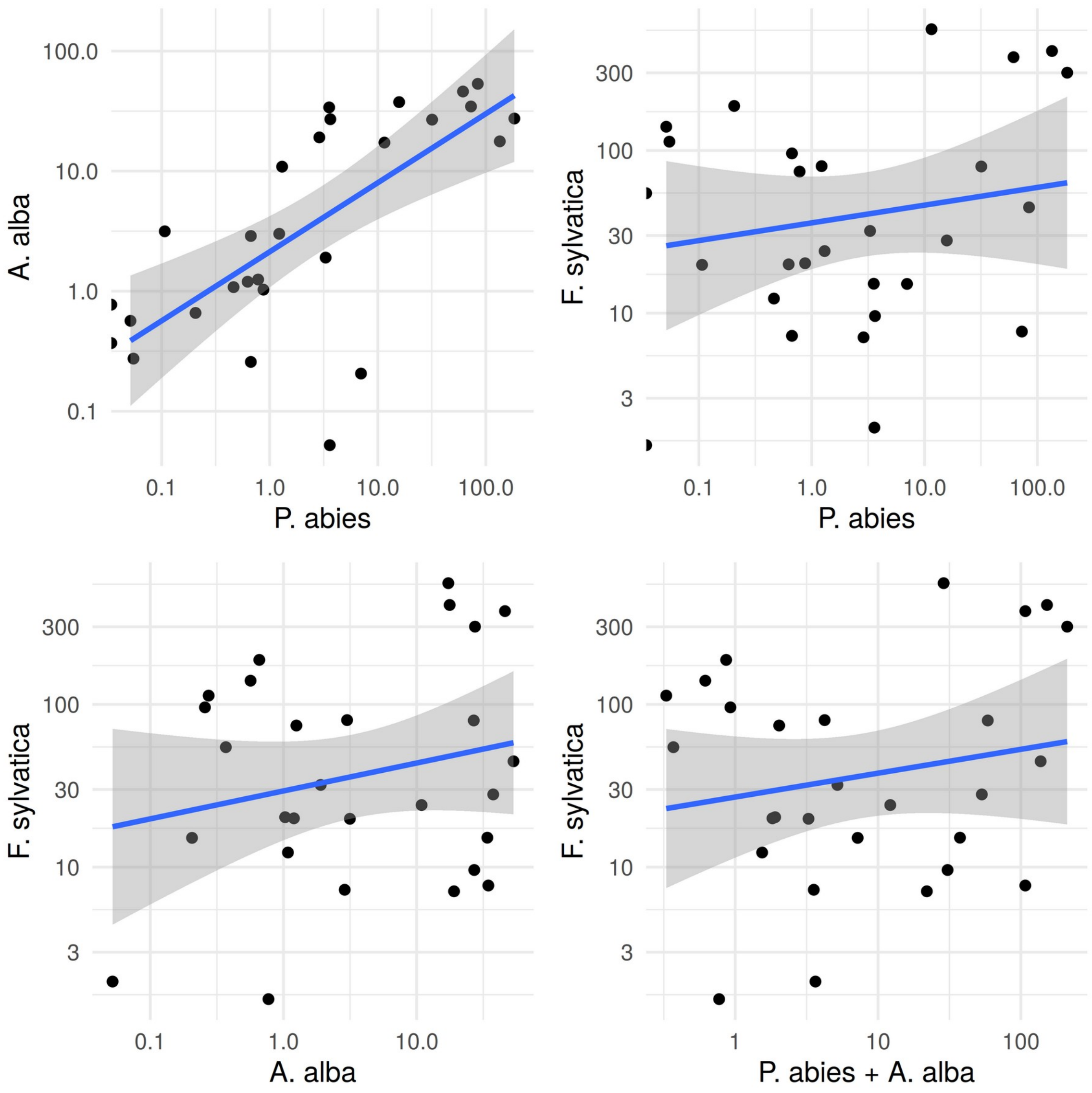

Figure S3.2: Pairwise visualisation of seed rain for the dominant tree species on log-scale (smoothed conditional means with a linear regression as smoothing method were added for visual support). Spearman rank correlation was significant and moderate to strong between conifers (*A. alba* and *P. abies*; Spearman rho = 0.69 p = 4.5e-05, n = 28, topleft) but not for the other combinations.

Appendix S4: Temperature estimation via R-package Microclima

We estimated hourly values for aboveground temperature at each study site by using the function ‚runauto‘ from the R-package ‚microclima‘ (Version 0.1; Maclean et al., 2019). We prepared polygons of the study sites and added a buffer of 50 m around each site. Subsequently, we used these polygons to clip a digital terrain model with a spatial resolution of 30 m which then served as input for the function ‚runauto‘. We specified the habitat type for each site as follows: managed forest MFb as ‘Evergreen needleleaf forest’, primeval forests PFb and PFs as ‘Deciduous broadleaf forest’, the windthrow site WTs as ‘Closed shrublands’ and the avalanche site AV as ‘Open shrublands’. We ran the function for each site and each year separately and obtained estimated temperature values at a height of 0.1 m aboveground with a spatial resolution according to our digital terrain model at an hourly interval. We converted the timezone from Coordinated Universal Time into Central European Time and aggregated the temperature values for each timestep and site to obtain an hourly mean temperature value for each site. We further summarized the mean temperatures between 7 pm and 6 am in order to obtain nightly temperature estimates that correspond to the time of the day when small mammal live traps where set active. The mean nightly temperature (hereafter referred to as MNT) estimates of the site WTs correlated strongly with corresponding MNT measurements obtained from a weatherstation nearby (Pearson $r = 0.94$, $p < 2.2e\text{-}16$, $n = 2830$). The estimated MNT was lower than the measured values in 96,5 % of all nights in our dataset. However, the difference was weak during the summer months (on average less than 3 °C from May to August) and more pronounced during the winter (median of temperature difference always below 10 °C; Figure S4.1). As we conducted small mammal trapping only between spring and autumn, we did not further investigate the systematic bias during the winter months.

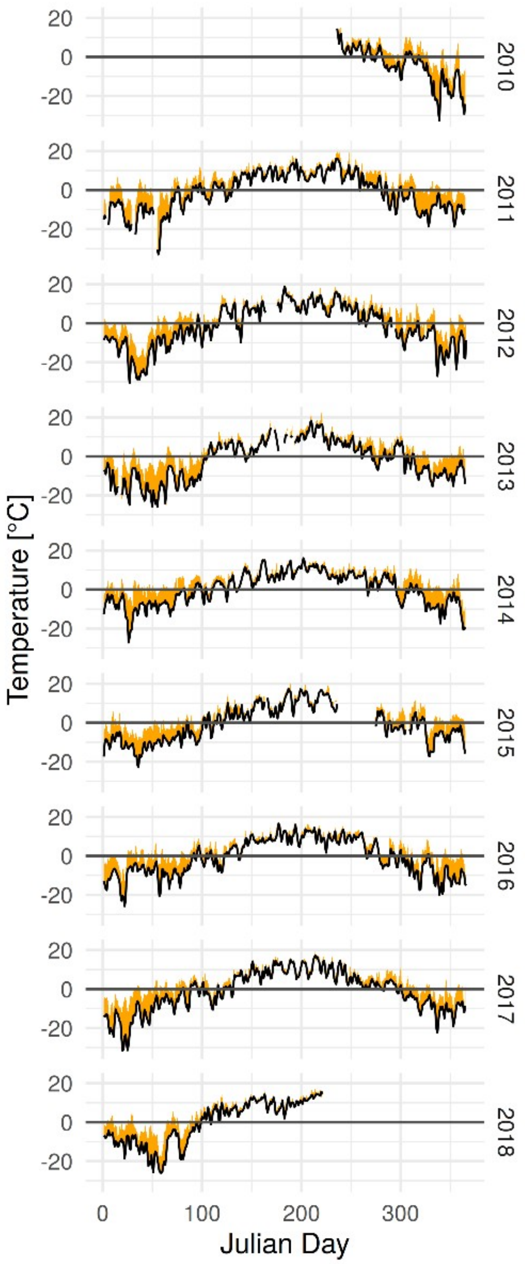

Figure S4.1: Mean temperature values for each night (7pm to 6am) categorized by year. The orange ribbon depicts the difference between the average of the measured temperature of the weather station and the estimated average temperature according to the microclimate model for the site WTs (black line). The site WTs is about 20 m apart from the weather station. For illustration purposes we removed estimated temperature for corresponding missing values from the weather station due to technical malfunction.

Appendix S5: Marginal effects on detection probability

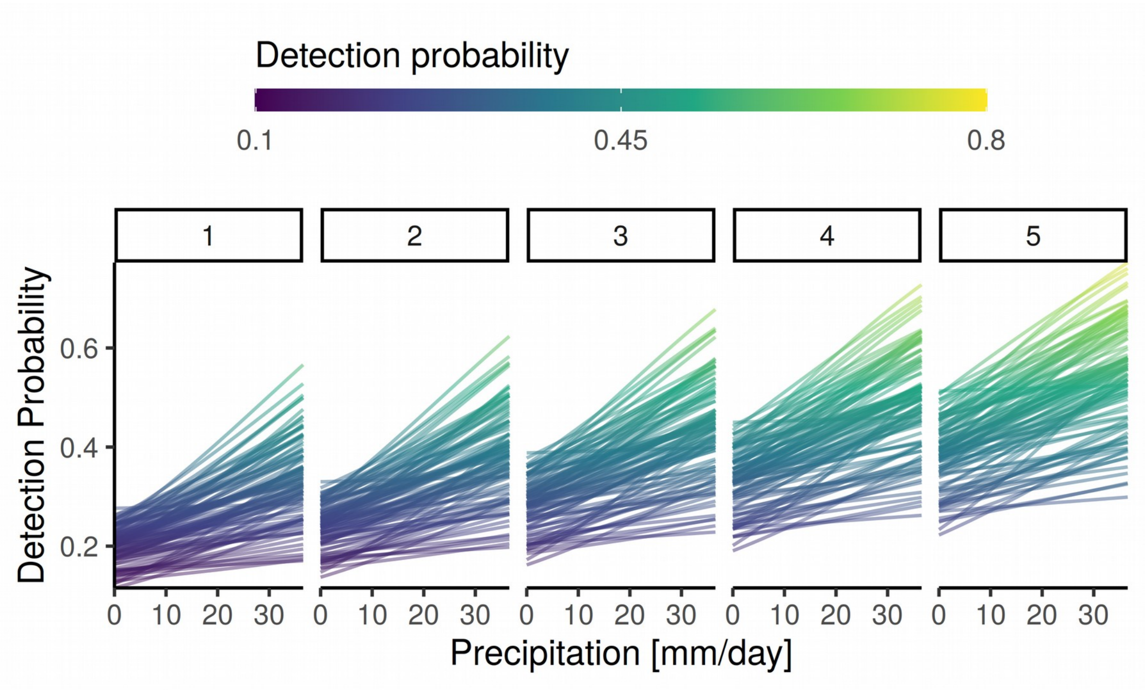

Figure S5.1: Marginal effects of precipitation and consecutive trap nights on detection probability ‘p’ of our N-mixture model for *Apodemus* spp. (each line depicts one of 100 random draws of the posterior predictions). The 95% credible interval of the posterior distribution does not include zero for both covariates. Other covariates are held constant at their mean value.

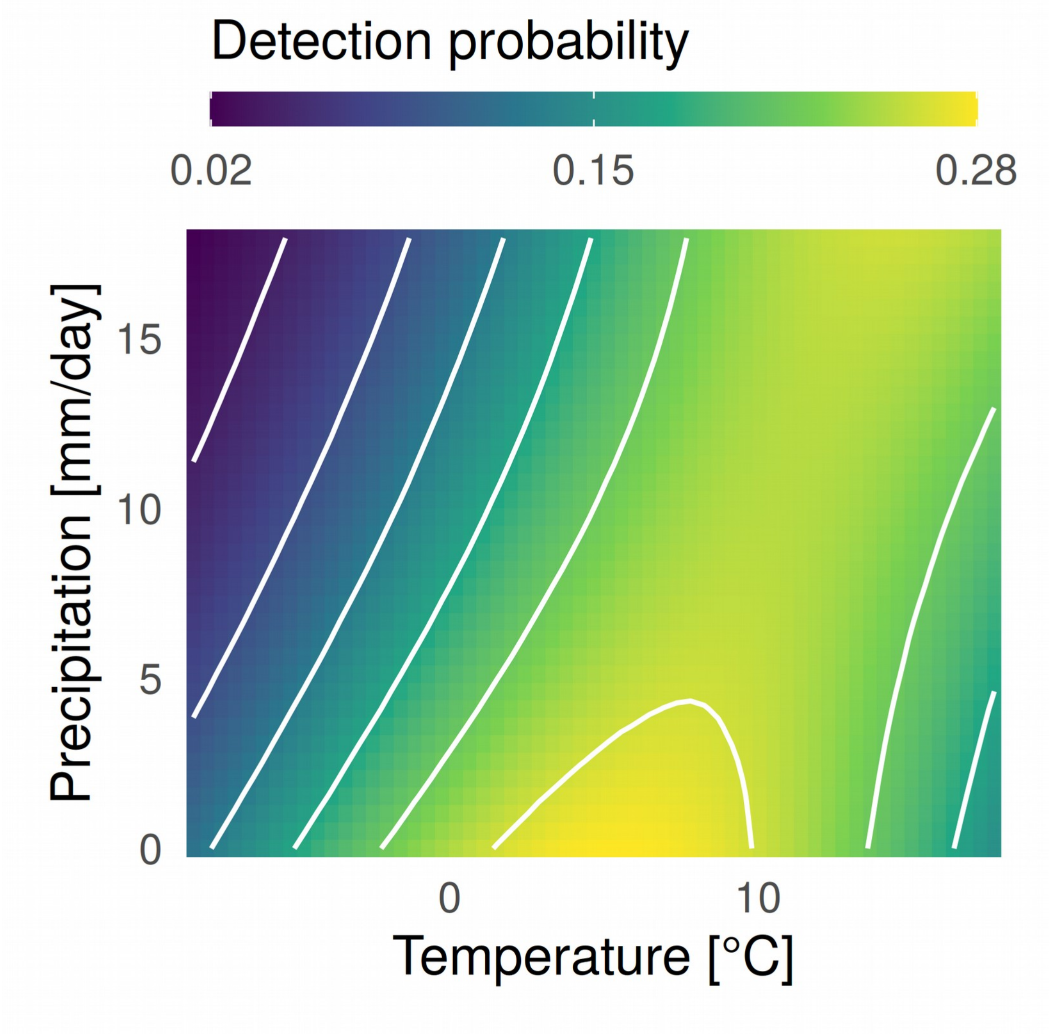

Figure S5.2: Marginal effects of temperature and precipitation on detection probability ‘p’ of our N-mixture model for *Myodes glareolus*. The color of each tile represents the arithmetic mean for detection probability, ranging from 0.02 to 0.28. The 95% credible interval of the posterior distribution does not include zero for the interaction between these two covariates. Other covariates are held constant at their mean value; trap night of the session was fixed at 1.

References

Maclean, I. M. D., Mosedale, J. R., & Bennie, J. J. (2019). Microclima: An r package for modelling meso- and microclimate. Methods in Ecology and Evolution, 10(2), 280–290. https://doi.org/10.1111/2041-210X.13093
